# Volatile Compound Profiling of Seven *Tuber* Species Using HS-SPME-GC-MS and Classification by a Chemometric Approach

**DOI:** 10.1021/acsomega.3c05292

**Published:** 2023-09-05

**Authors:** Cansu Korkmaz, Khaoula Hellal, Meltem Taş Küçükaydın, Fatih Çayan, Selçuk Küçükaydın, Mehmet Emin Duru

**Affiliations:** † Department of Biology, Faculty of Science, Muğla Sıtkı Koçman University, 48000 Muğla, Turkey; ‡ Department of Chemistry, Faculty of Science, Muğla Sıtkı Koçman University, 48000 Muğla, Turkey; § Department of Chemistry and Chemical Processing Technologies, Muğla Vocational School, Muğla Sıtkı Koçman University, 48000 Muğla, Turkey; ∥ Department of Medical Services and Techniques, Köyceğiz Vocational School of Health Services, Muğla Sıtkı Koçman University, 48800 Köyceğiz/Muğla, Turkey

## Abstract

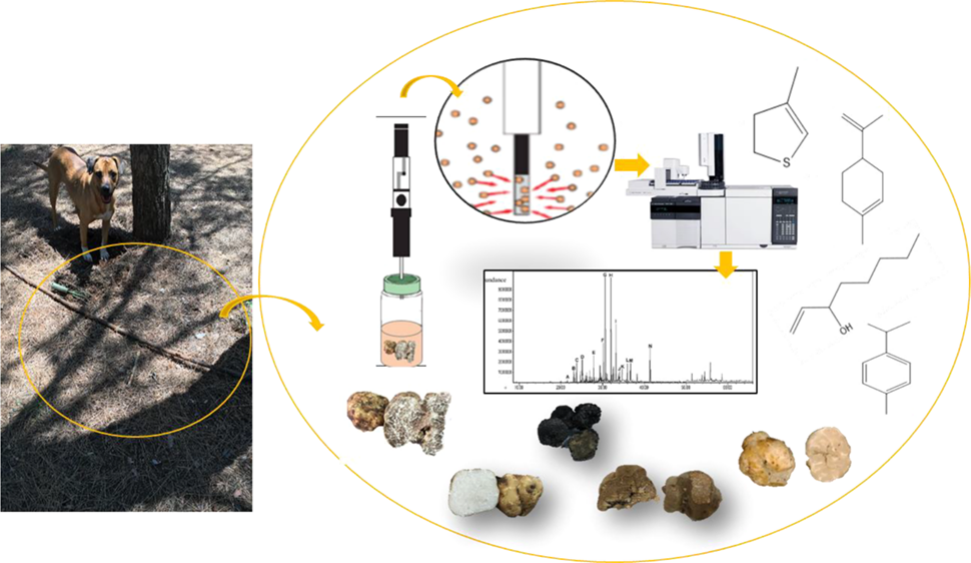

Edible mushrooms are important providers of nutrients and are well recognized for their particular organoleptic properties. The volatiles that *Tuber* releases serve purposes beyond simply appealing to our sense of smell. Truffles have different smells and tastes due to the fact that they contain different volatile components; therefore, aroma is essential in defining the organoleptic properties and quality of truffles. In this research, seven *Tuber* species, namely, *Tuber ferrugineum*, *Tuber nitidum*, *Tuber excavatum*, *Tuber rufum*, *Tuber puberulum*, *Tuber aestivum*, and *Tuber borchii* were selected. The primary objective of this study was to carry out the first in-depth investigation of the volatile compounds and chemometric analysis of seven truffle species from the *Tuber* genus that are grown in Turkey. The SPME headspace combined with GC–MS analysis identified 60 volatiles from different classes, with the abundance of terpenes being followed in a decreasing order by alcohols, aldehydes, sulfides, ketones, and other aromatic compounds. According to the chemometric analysis, methional, 3-methyl-4,5-dihydrothiophene, *p*-(methylthio) benzaldehyde, 3-octene, linalyl acetate, methyl caproate, and β-trans-ocimene could be highlighted as markers for *T. borchii* grown in Turkey. This investigation was conducted for the first time using *T. ferrugineum*, *T. puberulum*, and *T. nitidum*. The comparison of the volatile profile of these tubers’ species displayed branded differences. Thus, the knowledge gained from this research may pave the way to identify the key aroma contributors in the chosen *Tuber* species.

## 1 Introduction

Edible mushrooms are important providers of nutrients and are well recognized for their particular organoleptic properties. Therefore, they are considered as a premium gourmet ingredient and one of the most precious foodstuffs, including some mycorrhizal mushrooms, which are not commonly seen on the market but are prized seasonal delicacies for regional cuisine.^1,2^ Two categories can be used to classify edible fungi, epigeous carpophores commonly known as mushrooms, and hypogeous species that grow underground, known as truffles.^3^ The genus *Tuber* is one of the truffles that are most significant economically, among which are the species *Tuber ferrugineum* Vittad., *T. nitidum* Vittad., *T. excavatum* Vittad., *T. rufum* Pico., *T. puberulum* Berk. & Broome., *T. aestivum* Vittad., and *T. borchii* Vittad. renowned for their peculiar aroma. The ascomycetes contained in the genus *Tuber* spp. are ectomycorrhizal fungi that develop in symbiosis with the roots of numerous vascular plant species that are both Angiosperms and Gymnosperms. This fungus has an ascoma that is a hypogeous complex apothecium or truffle.^4^

There are currently between 180 and 220 different *Tuber* species in the globe, of which 30 are traded economically.^5^ The medical potential of these species is thought to be enormous, yet this is still an area that needs more research with regard to such properties. More recent studies have investigated the therapeutic potential of truffles and reported that they possess many bioactivities such as antiviral, antibacterial, antimutagenic, antioxidant, and anti-inflammatory properties.^3,4,6,7^ According to an analysis of their nutrient profile, truffles are a good source of both carbs and proteins. Minor constituents of truffles, including minerals, amino acids, and fatty acids, have often been identified. Other less significant chemical components, including phenolic compounds or tocopherols, have only been investigated just in two truffle species.^2,3,8^ As far as we know, the sensation we refer to as ″aroma″ is really a combination of inputs that our brain elaborates in a complicated way, combining the taste, sight, and smell of food in one perception.^5^ As one might anticipate, the volatiles that *Tuber* releases serve purposes beyond simply appealing to our sense of smell. Truffles have different smells and tastes due to the fact that they contain different volatile components, therefore aroma is essential in defining the organoleptic properties and quality of truffles.^9,10,12^ The properties of the soil, the surrounding environment, and particularly the host trees have a significant impact on the chemical makeup of truffles.^2^ Recent studies have used various methodologies, mainly HS-SPME-GC–MS, to analyze the volatile components of different truffle species.^5^ When the volatile compounds of truffles were analyzed at the level of the compound class, it was frequently found that alcohols, aldehydes, ketones, acids, esters, terpenes, and sulfur compounds were detected. According to previous investigations, 2-methylbutanal, 3-methylbutanal, dimethyl sulfide, dimethyldisulfide, 2-methyl-1-propanol, and 1-octen-3-ol are found to be the most common volatile compounds of truffles.^13^

Turkey is currently a crucial area for the investigation of truffles. Turkey is situated in the Mediterranean region, where the countries have a rich biodiversity of ectomycorrhizal fungi like truffles and it acts as a bridge between European and Asian flora. To date, the identification of 104 truffle taxa, including 35 genus and 20 families, has been conducted in Turkey.^14^ In this research, seven tubers, namely, *T. ferrugineum*, *T. nitidum*, *T. excavatum*, *T. rufum*, *T. puberulum*, *T. aestivum*, and *T. borchii* were selected. To the best of our knowledge, few studies that address the aromatic chemical composition of these seven Turkish *Tuber* species are available. Therefore, this study set out to conduct the first thorough investigation of the aromatic chemical, as well as chemometric analysis of seven truffle species belonging to the *Tuber* genera grown in Turkey. The ongoing quest for promising medication candidates is the primary motivation behind the investigation of bioactive secondary metabolites from Turkish species, which could have an ethnopharmacological importance. Despite the fact that the volatile organic compounds of many *Tuber* species grown in other countries have been examined, there is no research in the literature on the chemical composition and chemometric analysis of the volatile organic compounds of *Tuber* species grown in Turkey. A *Tuber* species’ economic worth on the market is determined by its fragrance. Therefore, identifying the volatile chemical compounds that give truffles their distinctive aroma is crucial for the commercialization of these species. The purpose of this study was to characterize the volatile organic compounds of seven *Tuber* species occurring naturally in Turkey. Additionally, the ultimate goal of this study is to establish correlations between species by conducting chemometric analyses of volatile organic compounds from seven different *Tuber* species.

## 2 Materials and Methods

### 2.1 Truffle Material

This study was carried out on seven *Tuber* samples collected from different areas of Turkey during the year 2020. The voucher specimens have been deposited at the fungarium of Natural Products Laboratory of Mugla Sıtkı Kocman University. All the samples were stored in hermetically closed glass bottles at 4 °C until the analysis. Table 1 lists the fungarium number, the tree species in which they are found as mycorrhizal, and the locations where these species are collected.

**Table 1:**
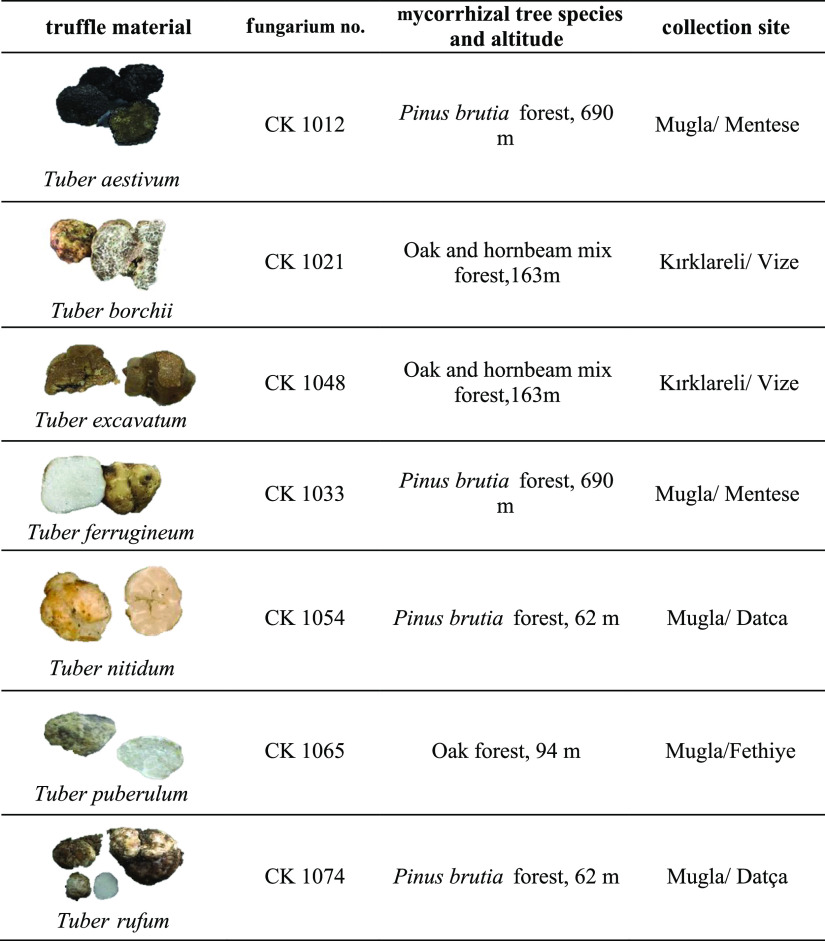
Fungarium Numbers and Collection Sites of the Selected *Tuber* Species

### 2.2 Extraction of the Volatile Compounds

To determine the volatile organic compounds of *Tuber* species in Turkey, truffles were freshly chopped and analyzed by the Headspace Solid Phase Microextraction-GC/MS (HS-SPME-GC/MS) system according to the method of Duru et al.^15^ Solid-phase microextraction (SPME) was followed by gas chromatography (GC) and gas chromatography–mass spectrometry (GC–MS) systems to analyze the volatile compounds in tuber samples. According to the manufacturer’s instructions, the fiber was preconditioned and thermally cleaned at the injection port of a gas chromatograph before analysis. For HS-SPME extraction, 5 g of tuber sample was dissolved in 5 mL of 20% sodium chloride solution and placed in a 20 mL amber glass vial and hermetically sealed. The vial was maintained in a water bath at 50 °C during equilibration (30 min) and extraction (50 min) and was partially submerged so that the liquid phase of the sample was in the water. All the experiments were performed under constant stirring velocity by a magnetic stirrer. After sampling, the SPME fiber was withdrawn into the needle, removed from the vial, and inserted into the injector (250 °C) of the GC and GC–MS for 6 min, where the extracted volatiles were thermally desorbed directly to the GC column.

### 2.3 GC–MS Analysis

GC–MS analysis was carried out using an ion trap MS spectrometer (Varian). The fused silica nonpolar capillary column Rxi-5Sil MS (Restek) (30 m × 0.25 mm I.D, film thickness 0.25 μm) was used in the GC–MS. Helium was used as the carrier gas in the chromatography with a flow rate of 1.4 mL/min. The injector and MS-transfer line were maintained at 250 and 270 °C, respectively. The initial column temperature was maintained at 60 °C for 5 min, then increased to 280 °C at a rate of 4 °C/min, and then remained at this temperature for 5 min. Thermal desorption of the compounds from the fiber coating took place in the GC injector at 200 °C for 15 min in splitless mode with a flow rate of 1.4 mL/min. The compounds were identified by matching their mass spectra with those in a mass spectrometry libraries (Wiley, ADAMS, and NIST 08 MS) and coinjection with standards (whenever possible), together GC retention indices determined using a homologous series of C_7_–C_30_ alkanes (Supelco), as well as by comparison of the fragmentation patterns of the mass spectra reported in the literature.^16^ The relative area of each compound was expressed as a percentage from the total chromatogram integration recorded and calculated with data from GC/MS analyses and manual peak integration without correction factor.

### 2.4 Statistical Analysis

Results were expressed as the average of three repeats and given as the mean ± S.E. MINITAB 16.0 software was used to perform all statistical computations for chemometric investigations of the volatile chemicals in tuber samples using principal component analysis (PCA) and hierarchical cluster analysis (HCA). With the Ward Linkage approach and Euclidean distance, hierarchical relations were obtained using cluster analysis. The correlations of volatile compounds were performed by Pearson’s correlation by using SPSS v22.0 software.

## 3 Results and Discussion

### 3.1 Volatile Metabolites and Their Contribution to the Aroma of Tubers

Seven different *Tuber* samples were investigated using headspace solid-phase microextraction (HS-SPME), GC–MS, and the results were used to generate the first comprehensive patterns of headspace volatile chemicals of the selected *Tuber* species. A total of 60 distinct compounds were identified in the *Tuber* samples that were selected for this investigation. As listed in Table 2, the detected compounds are a blend of alkanes and alkenes, sulfur compounds, alcohols, esters, aldehydes, ketones, aromatic compounds, and terpenes. It is noteworthy to highlight that the volatile constituents of tubers from a certain species vary significantly. Also, correlations of volatile compounds of truffles by Pearson’s correlation test are given in Table S1. *Tuber* species have an earthy, cheese-like, pungent, garlicky, leathery, vanilla-like, dusty, creamy, and even gasoline-like aromas that range in strength from mild to intense.^10^ Volatile profiles of tubers can differ and fluctuate due to the biotic variables (bacteria, fungus, yeasts, mesofauna, and host plant) that frequently coexist in tubers land, in addition to the abiotic factors (soil characteristics, rainfall and temperature, microclimatology, and mycelia connectivity).^11,17^

**Table 2:** Volatile Organic Compounds of Seven *Tuber* Species in Turkey (%)^a^

class of compounds	compounds	LRI^b^	RI^c^	ID^d^	*T.aestivum* (%)	*T.borchii* (%)	*T.excavatum* (%)	*T.ferrugineum* (%)	*T.nitidum* (%)	*T.puberulum* (%)	*T.rufum* (%)
sulfur compounds	methional	870	872	RI, MS		10.64 ± 0.18					
	3-methyl thioprophanal	879	885	RI, MS	6.23 ± 0.12						
	5-methyl benzothiophene	1075	1077	RI, MS				0.37 ± 0.04	0.80 ± 0.11		
	3-methyl-4,5-dihydrothiophene	1128	1125	RI, MS		30.68 ± 0.54					
	2-methyl, 4-propylthiazole	1280	1277	RI, MS					1.42 ± 0.14		
	*p*-(methylthio) benzaldehyde	1340	1346	RI, MS		1.86 ± 0.17					
alkanes and alkenes	3-octene	801	799	RI, MS		1.55 ± 0.11					
	hexadecane	1615	1612	RI, MS				0.34 ± 0.01	0.92 ± 0.06		
alcohols	1-octen-3-ol	958	961	Co-GC, MS, RI		12.67 ± 0.24	9.84 ± 0.21	1.87 ± 0.13	1.72 ± 0.05	30.03 ± 0.51	
	3-octanol	994	992	Co-GC, MS, RI	0.48 ± 0.02		22.62 ± 0.37			0.67 ± 0.08	
	2-octen-1-ol	1028	1024	Co-GC, MS, RI	6.11 ± 0.08						
	3-octen-1-ol	1061	1062	Co-GC, MS, RI	0.51 ± 0.05						
	octanol	1066	1068	Co-GC, MS, RI			4.89 ± 0.14		0.77 ± 0.10		
	nonanol	1088	1089	Co-GC, MS, RI		5.28 ± 0.14	2.86 ± 0.08	6.88 ± 0.23	9.27 ± 0.25	0.39 ± 0.05	0.68 ± 0.07
	(E)-3-decen-1-ol	1230	1232	Co-GC, MS, RI		0.49 ± 0.05	0.98 ± 0.10	3.43 ± 0.08	4.90 ± 0.17	0.32 ± 0.05	0.36 ± 0.04
	hexadecanol	1821	1825	MS, RI				0.16 ± 0.02	1.89 ± 0.05		
esters	ethyl phenylacetate	1213	1212	Co-GC, MS, RI	6.04 ± 0.16						4.22 ± 0.10
	linalyl acetate	1245	1242	Co-GC, MS, RI		0.23 ± 0.02					
	bornyl acetate	1277	1275	Co-GC, MS, RI		0.45 ± 0.11		0.15 ± 0.03	1.92 ± 0.22		
	methyl caproate	1310	1313	MS, RI		0.38 ± 0.14					
	methyl myristate	1700	1708	Co-GC, MS, RI				0.39 ± 0.07			
aldehydes	hexanal	845	847	Co-GC, MS, RI			2.16 ± 0.11	0.78 ± 0.10	0.53 ± 0.07	0.65 ± 0.11	
	heptanal	872	880	Co-GC, MS, RI		1.98 ± 0.05	1.06 ± 0.09	2.75 ± 0.15	0.37 ± 0.02	1.19 ± 0.03	
	2-methylene-hekzanal	890	892	MS, RI	1.67 ± 0.04		1.21 ± 0.12	4.80 ± 0.16	0.96 ± 0.05	0.65 ± 0.09	1.73 ± 0.05
	*cis*-2-heptenal	933	935	MS, RI	0.95 ± 0.06	0.63 ± 0.12	2.70 ± 0.16	0.76 ± 0.08	0.64 ± 0.05	0.43 ± 0.03	
	octanal	982	983	Co-GC, MS, RI	1.18 ± 0.10	3.09 ± 0.24	0.65 ± 0.10	2.62 ± 0.20	2.53 ± 0.04	0.91 ± 0.08	
	[E]-2-octenal	1035	1039	Co-GC, MS, RI	5.07 ± 0.20	6.53 ± 0.18	5.27 ± 0.29		12.06 ± 0.11	1.58 ± 0.14	
	[E]-2-nonenal	1130	1133	MS, RI			1.74 ± 0.14	0.35 ± 0.07	2.25 ± 0.08		
	[E,E]-2,4-nonadienal	1210	1211	MS, RI			0.48 ± 0.05	0.05 ± 0.01	0.56 ± 0.16		
	*cis*-2-decenal	1251	1250	MS, RI		0.16 ± 0.07					
	[E,E]-2,4-decadienal	1288	1292	MS, RI			2.05 ± 0.10				
	5-methyl-2-phenyl-2-hexenal	1489	1493	MS, RI		0.35 ± 0.05					
ketones	6-methyl-5-heptene-2-one	962	966	MS, RI			0.48 ± 0.12	3.29 ± 0.14	3.94 ± 0.25		0.58 ± 0.04
	3-octanon	988	988	Co-GC, MS, RI	1.86 ± 0.12	3.05 ± 0.10	16.44 ± 0.22			6.55 ± 0.15	
	3-octen-2-one	1032	1036	Co-GC, MS, RI			5.13 ± 0.24				
	1-(2,8,8-trimethyl-5,6,7,8-tetrahydro-4H-cycloheptafuran-5-yle) ethanone	1631	1632	MS, RI		0.14 ± 0.06					
aromatic compounds	styrene	860	862	MS, RI		0.92 ± 0.10	1.16 ± 0.05				
	benzaldehyde	955	956	Co-GC, MS, RI	10.65 ± 0.24	0.67 ± 0.08		0.76 ± 0.10	0.37 ± 0.04		0.72 ± 0.13
	phenyl acetaldehyde	1044	1043	Co-GC, MS, RI	0.52 ± 0.08	6.05 ± 0.17		0.67 ± 0.15	1.61 ± 0.17		0.82 ± 0.06
	acetophenone	1055	1058	Co-GC, MS, RI		0.31 ± 0.05					
	*p*-hydroxy thioanisole	1261	1265	MS, RI					4.67 ± 0.20		
terpenes	α-pinene	927	928	Co-GC, MS, RI	0.75 ± 0.08	0.29 ± 0.05		0.53 ± 0.05			0.79 ± 0.03
	β-pinene	963	963	Co-GC, MS, RI	4.57 ± 0.19			1.02 ± 0.18	0.64 ± 0.06		8.78 ± 0.24
	β-myrcene	991	993	Co-GC, MS, RI				2.30 ± 0.11	1.07 ± 0.12		1.77 ± 0.15
	*p*-cymene	1025	1027	Co-GC, MS, RI	5.22 ± 0.31	0.39 ± 0.09	13.08 ± 0.44	7.54 ± 0.21	4.57 ± 0.32	6.98 ± 0.24	5.91 ± 0.22
	limonene	1032	1032	Co-GC, MS, RI	38.84 ± 0.67	0.42 ± 0.14		50.11 ± 0.75	30.41 ± 0.45	49.65 ± 0.58	61.26 ± 0.70
	eucalyptol	1034	1035	Co-GC, MS, RI		5.62 ± 0.22	4.06 ± 0.31				
	β*-trans*-ocimene	1045	1047	Co-GC, MS, RI		2.76 ± 0.08					
	γ-terpinene	1047	1048	Co-GC, MS, RI				6.59 ± 0.26			5.92 ± 0.36
	dihydro myrcenol	1075	1074	MS, RI					2.06 ± 0.16		
	β-linalool	1082	1083	Co-GC, MS, RI		1.66 ± 0.15	0.85 ± 0.12	0.37 ± 0.03	1.35 ± 0.04		
	borneol	1134	1134	Co-GC, MS, RI		0.25 ± 0.07					
	α-terpineol	1172	1175	Co-GC, MS, RI				0.24 ± 0.05	1.19 ± 0.12		
	thymol	1275	1278	Co-GC, MS, RI	0.61 ± 0.06	0.12 ± 0.01					
	carvacrol	1288	1290	Co-GC, MS, RI	6.75 ± 0.13	0.15 ± 0.03					6.46 ± 0.18
	α-terpinyl acetate	1330	1334	Co-GC, MS, RI		0.12 ± 0.06					
	α-himachalen	1411	1414	Co-GC, MS, RI	0.61 ± 0.08						
	geranyl acetone	1425	1429	Co-GC, MS, RI		0.11 ± 0.02	0.29 ± 0.07	0.88 ± 0.02	2.29 ± 0.08		
	selinane	1431	1432	MS, RI					2.32 ± 0.10		
others	2-indanone	1127	1128	MS, RI	1.38 ± 0.11						
total					100.00	100.00	100.00	100.00	100.00	100.00	100.00

a Percentage concentration.

b Literature retention index.

c Retention index on the Rxi-5Sil MS-fused silica column.

d Identification, Co-GC: coinjection, based on comparison with reference compounds, MS: based on comparison with WILEY, ADAMS, and NIST 08 databases.

#### 3.1.1 Terpenes

Terpenes are the scent chemicals that are most prevalent in almost all the seven tubers selected in this investigation. Hence, they seem to play important roles in tubers aroma. According to Table 1, limonene was found to be the most abundant compound in *T. rufum* (61.26%), *T. ferrugineum* (50.11%), *T. puberulum* (49.65%), *T. nitidum* (30.41%), *T. aestivum* (38.84%), and *T. borchi* (0.42%). Based on the values listed in Table 1, limonene does appear, however, to be a marker of aroma since it is included in all the tubers other than *T. excavatum*. In addition to limonene, *p*-cymene was detected mostly in *T. excavatum* with a value of (13.08%), followed by (7.54%) in *T. ferrugineum* and (6.98%) in *T. puberulum*. On the other hand, *T. aestivum* and *T. rufum* were found to contain a good amount of carvacrol, with values of (6.75%) and (6.46%), respectively. Back to the literature review, it is noteworthy to highlight that limonene was detected previously in *T. magnatum*, *T. brumale*, *T. sinensis*, *T. sinoalbidum*, and *T. sinoexcavatum*; however, *p*-cymene was reported only in *T. magnatum*.^2,10,18−20^ Therefore, these two major terpenes are the first time to be reported in the selected tubers of this study. Compounds such as eucalyptol, γ-terpinene, geranyl acetone, and other terpenes were also detected in different tubers with different values.

#### 3.1.2 Aldehydes

After terpenes, aldehydes have amounted to the second prevalent volatile class found in the analyzed tubers. *T. nitidum* exhibited the highest value of [E]-2-octenal with a value of (12.06%). Among the aldehydes detected, [E]-2-octenal was considered as an important contributor to the aroma in all tubers except *T. ferrugineum* and *T. rufum* where [E]-2-octenal could not be found. Aldehydes such as 2-methylene-hekzanal, *cis*-2-heptenal, and octanal were detected almost in all seven tubers. However, *cis*-2-decenal, [E,E]-2,4-decadienal, 5-methyl-2-phenyl-2-hexenal, and [E,E]-2,4-nonadienal are aldehydes that were found with trace levels or not found in all the tubers.

#### 3.1.3 Alcohols

1-Octen-3-ol, commonly recognized as mushroom alcohol, is a major aroma determinant of fungal fragrance with earthy flavor derived from fatty acid oxidation.^11^ In *T. puberulum* and *T. borchii*, 1-octen-3-ol was the abundant alcohol, showing a presence of 30.03 and 12.67%, respectively. These findings are in line with those reported previously by Lee et al. and Mustafa et al.^8,10^ In addition to these two *Tuber* species, 1-octen-3-ol was also emitted by *T. excavatum* (9.84%) and by *T. ferrugineum* and *T. nitidum* with a lower percentage (>2%). On the other hand, 3-octanol represented the main alcohol in *T. aestivum* by a percentage of 22.62%; however, it was detected with a trace amount in *T. puberulum* and *T. aestivum*. Nonanol and (E)-3-decen-1-ol were presented in all the studied tubers, which are classified as white tubers and were clearly absent in the black tuber represented by *T. aestivum*.

#### 3.1.4 Sulfur Compounds

Overall, six sulfides have been reported, with an average of three compounds in *T. borchii*, two compounds in *T. nitidum*, and one compound in *T. aestivum* and *T. ferrugineum*. The tuber species *T. puberulum* and *T. rufum* did not exhibit any presence of sulfur compounds. Starting by *T. borchii*, which represented the higher sulfide content among the studies species with the existence of three compounds, namely, 3-methyl-4,5 dihydrothiophene, methional, and *p*-(methylthio) benzaldehyde with a presence of 30.68, 10.64, and 1.86%, respectively. Next, the conducted analysis exhibited the appearance of 5-methyl benzothiophene and 2-methyl, 4-propylthiazole in *T. nitidum* species with values of 0.80 and 1.42%. Finally, 3-methyl thioprophanal was detected in *T. aestivum* and recorded an acceptable value of 6.23%, while 5-methyl benzothiophene was detected in *T. ferrugineum* with a lower value (0.37%). Due to their distinctive notes and extremely low olfactory thresholds for the human nose, sulfur compounds are known to make important contributions to the truffle aroma.^13^ Sulfur compounds 2-methyl-4,5-dihydrothiophene, bis(methylthio)methane, and 1-methylthio-1-propene were indicated as characteristic volatiles of *T. borchii*, *T. magnatum*, and *T. canaliculatum*, respectively, in earlier publications.^18,21^

#### 3.1.5 Ketones

Overall, only few ketones were identified in the tubers selected for this investigation. In *T. excavatum*, ketones were the most prevalent class, with 3-octanone accounting for 16.44% of all ketone compounds. In addition to *T. excavatum*, 3-octanone was found also as the only ketone in *T. puberulum* (6.55%) and *T. aestivum* (1.86%), and in *T. borchii* (3.05%) coupled with traces of 1-(2,8,8-trimethyl-5,6,7,8-tetrahydro-4H-cycloheptafuran-5-yle) ethanone. Bozok et al. previously declared that 3-octanone was found to be a primary volatile compound in straw and oyster mushrooms and known for its fruity, sweet, and lavender-like scent.^22^ Next, 3-octen-2-one, which exhibited a peppermint-like scent, was the second most abundant ketone in *T. excavatum* at 5.13% followed by 6-methyl-5-heptene-2-one with a lower value of 0.48%. Moreover, 6-methyl-5-heptene-2-one is another ketone that has been exhibited by *T. ferrugineum* at 3.29% and *T. nitidum* at 3.49%, and at very low levels by *T. rufum* at 0.58% *andT. aestivum* at 0.48%.

#### 3.1.6 Aromatic Compounds

In total, five aromatic compounds were successfully detected in all the tuber species except *T. puberulum*, which exhibited none of those compounds. Benzaldehyde was quantified in *T. aestivum* in a good amount (10.65%) compared to the very low amounts found in the rest of the tubers. In addition, phenylacetaldehyde hit its highest amount in *T. borchii*, recording a value of (6.05%). Unlike *p*-hydroxy thioanisole, which was exhibited with a notable amount only by *T. nitidum* (4.67%), acetophenone was found with a very little amount only in *T. borchii* (0.14%).

#### 3.1.7 Esters

Among the volatile compounds detected and identified in the present work, there were five esters, namely, ethyl phenylacetate, linalyl acetate, bornyl acetate, methyl caproate, and methyl myristate. By taking the notable amounts into consideration, ethyl phenylacetate recorded the highest amount among all the esters, and it was identified in *T. aestivum* at a value of 6.04% and in *T. rufum* at a value of 4.22%. The rest esters were detected at very low levels that did not pass (0.5%). The findings also revealed the absence of any esters in *T. puberulum* and *T. excavatum*.

#### 3.1.8 Alkanes/Alkenes and Others

Alkanes and alkenes, represented by 3-octene and hexadecane, were found in minute amounts in three species of tubers. Hexadecane was detected in *T. ferrugineum* and *T. nitidum* at values of 0.34 and 0.992%, respectively. However, 3-octene has been revealed in *T. borchii* at a higher value of 1.55%. On the other hand, other compounds such as 2-indanone were solely recognized in *T. aestivum* with a concentration of 1.38%.

**Figure 1:**
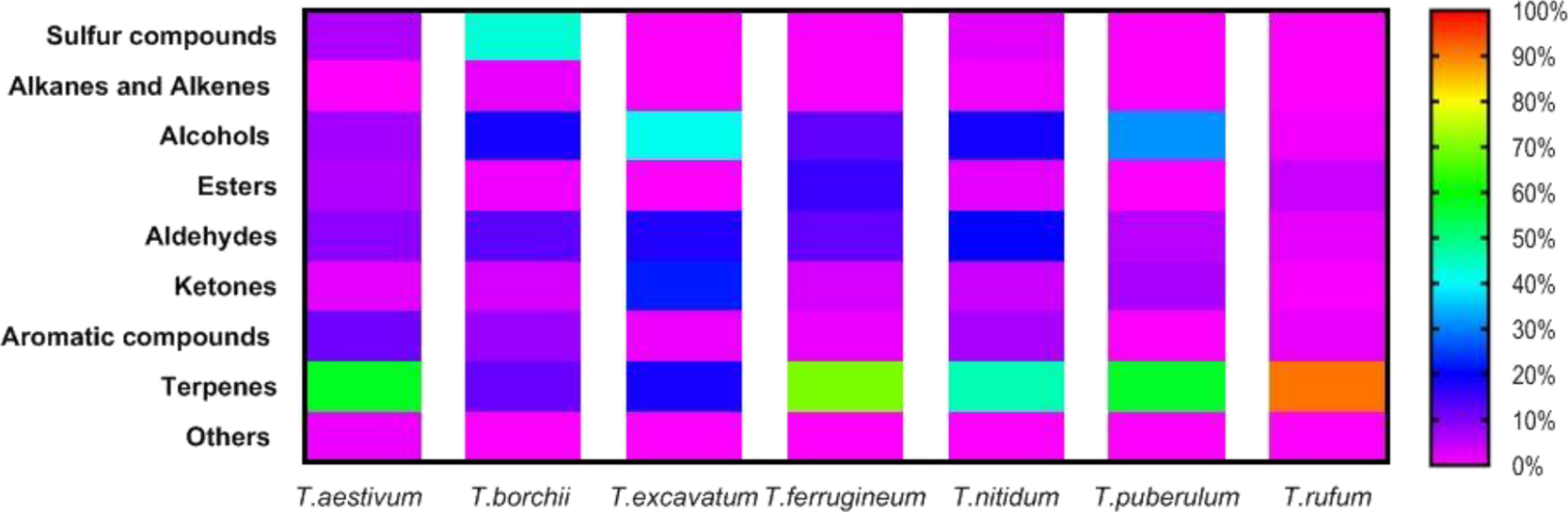
Relative percentages of volatile compound classes in truffle species identified by HS-SPME/GC–MS analysis.

The SPME headspace analysis identified 60 volatiles from different classes, with the abundance of terpenes being followed in a decreasing order by alcohols, aldehydes, sulfides, ketones, and other aromatic compounds. Figure 1 illustrates the relative percentages of volatile compound classes in tuber species identified by HS-SPME/GC–MS analysis. The aroma of tubers can range from mild to intense; the aroma profiles of tubers like *T. aestivum*, *T. borchii*, and *T. rufum* have previously been investigated.^2,10,18−20^ For instance, it has been suggested that *T. aestivum* (summer truffle) is less aromatic than *T. borchii* and *T. rufum*, which may help to explain why the aromatic chemicals in those species differ from those in *T. aestivum* and *T. rufum*.^23^ In *T. borchii*, sulfur compounds were discovered to predominate (more than 40% of the total aromatic compounds examined), which may account for its powerful garlicky scent. The terpene level in *T. aestivum* (≃57%) and *T. rufum* (≃90%) is likely what gives those tubers their fruity smells, though. However, *T. excavatum*, which was reported previously to have a potent, repulsive, pungent smell that smells like car paint, was found to possess a high level of alcohol (≃41%), mainly 3-octanol.^10,17^ Data from the literature indicate that our investigation addressed the volatile profile for the first time using Turkish *Tuber* species. Moreover, this investigation was conducted for the first time using *T. ferrugineum*, *T. puberulum*, and *T. nitidum*. In a nutshell, the comparison of the volatile profile of these tubers’ species displayed branded differences. Despite the variability in their volatile profile, truffles of a given species share common volatile compounds that can act as a species-specific fingerprint.^17^ This variation in their aroma profile could be referred to abiotic factors (such as soil characteristics, rainfall and temperature, microclimatology, and mycelia connectivity) and biotic factors (such as bacteria, fungi, yeasts, mesofauna, and host plant) that frequently covary in tubers.^24^ Each of the aforementioned factors has a maximum influence on how a particular species’ distinctive scent is shaped.

### 3.2 Chemometric Analysis

**Figure 2:**
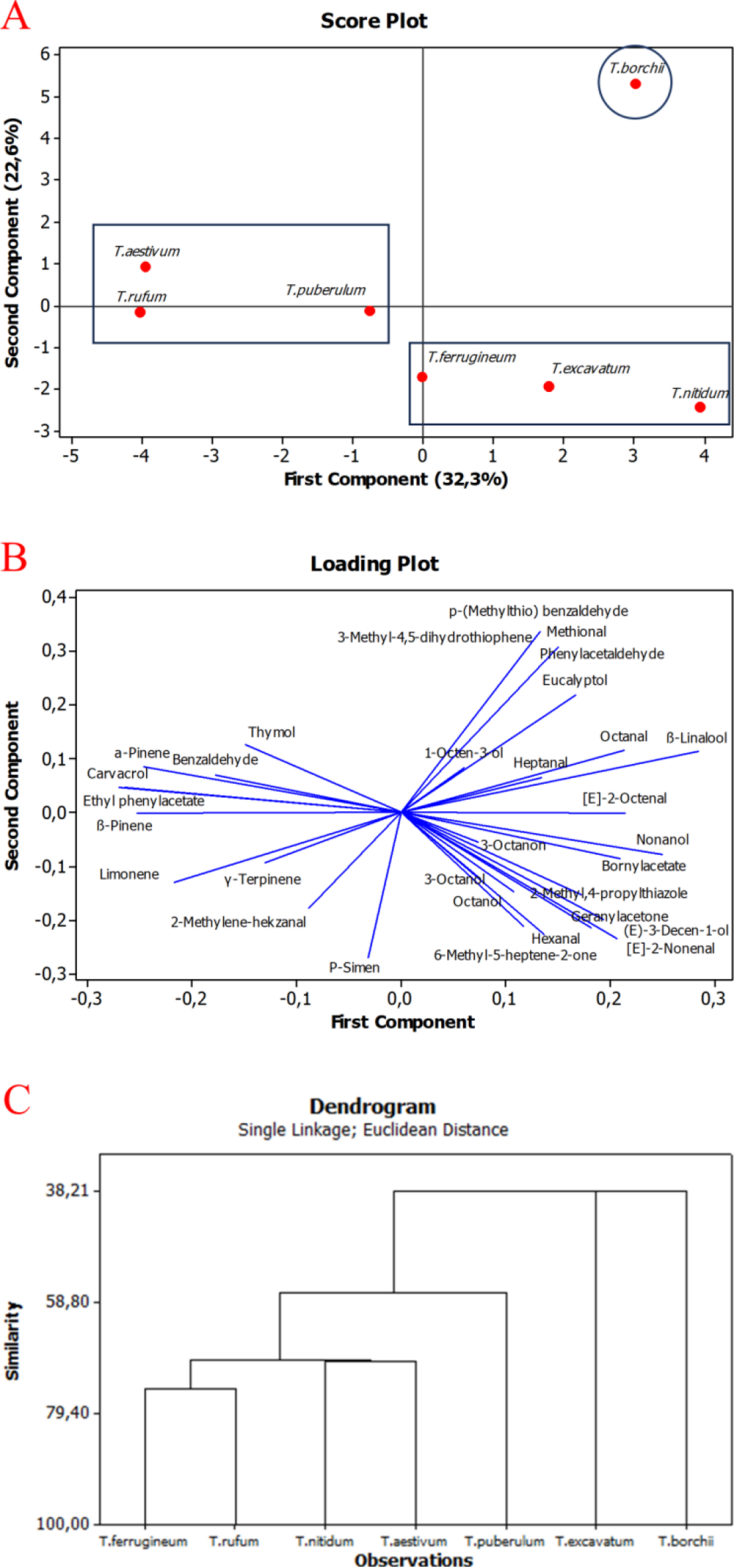
(A) Score plot graphic in terms of PC1 and PC2, (B) loading plot graphic in terms of PC1 and PC2 in *Tuber* species, and (C) dendrogram results obtained by the Euclidean distance and Ward Linkage method.

Chemometric analysis tools were used to better assess volatile variance within tuber specimens and highlight markers for each species. Based on the entities in Table 2, a PCA analysis was conducted, and the results are shown in Figure 2. As shown in Figure 2A, the first principal component (PC1) had already explained 32.3% of the total variance, whereas the second principal component (PC2) accounted for 22.6%. *T. borchii* clearly separated itself from the other tuber species, and it seems that *T. excavataum* also formed a close cluster to *T. borchii*. This observation might be explained by the fact that they were collected from the same location where *T. excavataum* forms ectomycorrhizae in the same host trees, which were oak and hornbeam trees. On the other hand, *T. rufum*, *T. puberulum*, *andT. aestivum* species are clustered close to one another. Finally, *T. ferrigineum*, *T. excavatum*, and *T. nitidum* separated together from the other species. These results were consistent with the results proposed by the loading plot in Figure 2B. On the one hand, compounds such as methional, 3-methyl-4,5-dihydrothiophene, *p*-(methylthio) benzaldehyde, 3-octene, linalyl acetate, methyl caproate, and β-trans-ocimene were suggested to be responsible for the dissimilarity in aromatic content between *T. borchii* and the rest of the tuber species, and on the other hand, these compounds could be highlighted as markers for *T. borchii* grown in Turkey. To identify the similarities and differences among the truffle samples, HCA was employed, and the resulting dendrogram is shown in Figure 2C. The Ward’s Linkage approach was used to create clusters, and the Euclidean distance was used to determine sample similarities. Figure 2C clearly indicates that the contents of *T. rufum*, *T. nitidum*, *T. aestivum*, and *T. ferrugineum* were very similar due to the existence of the same metabolites with different magnitudes. The data expressed by PCA and that proposed by the dendrogram for the seven tuber species, which was examined using HCA, are interrelated and complementary to each other.

## 4 Conclusions

More than 200 volatile compounds have been described in different tuber species as a result of earlier investigations that concentrated on screening and identifying volatile organic compounds. However, the amount of information regarding the quantities of fragrance molecules released by specific tubers growing in Turkey is still fairly limited in the scientific literature. The volatile compounds found in *Tuber* species increase interest in them and have a direct impact on how important they are commercially. For this reason, scientific studies on the determination of the volatile components of *Tuber* species and revealing the differences between species have increased in recent years. In this study, the volatile constituents of seven different *Tuber* species naturally distributed in Turkey were determined, and the species were compared in terms of volatile organic compounds by chemometric analyses. In order to better understand the volatile compounds of *T. excavatum*, *T. puberulum*, *T. aestivum*, *T. borchi*, *T. ferrugineum*, *T. rufum*, and *T. nitidum*, solid-phase microextraction (SPME), which is typically followed by gas chromatography–mass spectrometry, has been used to comprehensively profile and identify 60 volatiles from various classes with an abundance of terpenes following by alcohols, aldehydes, sulfides, ketones, aromatics, and esters. Interestingly, chemometric analysis revealed that volatiles including methional, 3-methyl-4,5-dihydrothiophene, *p*-(methylthio) benzaldehyde, 3-octene, linalyl acetate, methyl caproate, and β-trans-ocimene could be markers for *T. borchii* grown in Turkey, since results showed that *T. borchii* clustered separately than other studied tubers. To the best of our knowledge, this fingerprint profile is the first extensive study of aromatic metabolites from *T. ferrugineum*, *T. puberulum*, and *T. nitidum*, which may serve as the foundation for a framework that predicts the impact of various growing conditions on the aroma profile of these *Tuber* species. More research is required to determine how to use the knowledge of tubers’ fragrance components and incorporate them into goods with added value that are either directly related to tubers or that contain them.

## Supporting Information Available

Correlation analysis of volatile compounds of truffles by Pearson’s correlation test (PDF)
