# Supplementary material for: Volatile Compound Profiling of Seven Tuber Species Using HS-SPME-GC-MS and Classification by a Chemometric Approach
Source: ACS Omega. 2023 Sep 5;8(37):34111–9. doi: 10.1021/acsomega.3c05292 (PMC10515357; doi:10.1021/acsomega.3c05292)
Supplement: Supplementary file 1 — ao3c05292_si_001.pdf [file ao3c05292_si_001.pdf]

Volatile Compounds Profiling of Seven *Tuber* Species Using HS-SPME-GC-MS and Classification by Chemometric Approach

Cansu Korkmaz[1], Khaoula Hellal[2], Meltem Taş Küçükaydın[2], Fatih Çayan[3], Selçuk Küçükaydın[4]*, Mehmet Emin Duru[2]

[1] *Department of Biology, Faculty of Science, Muğla Sıtkı Koçman University, 48000, Muğla, Turkey*

[2] *Department of Chemistry, Faculty of Science, Muğla Sıtkı Koçman University, 48000, Muğla, Turkey*

[3] *Department of Chemistry and Chemical Processing Technologies, Muğla Vocational School, Muğla Sıtkı Koçman University, 48000 Muğla, Turkey*

[4] *Department of Medical Services and Techniques, Köyceğiz Vocational School of Health Services, Muğla Sıtkı Koçman University, 48800, Köyceğiz/Muğla, Turkey*

Supplementary information

*Corresponding author E-mail: selcukkucukaydin@mu.edu.tr

Table S1. Correlations of volatile compounds of truffles[a]

	**1**	**2**	**3**	**4**	**5**	**6**	**7**	**8**	**9**	**10**	**11**	**12**	**13**	**14**	**15**	**16**	**17**	**18**	**19**	**20**	**21**	**22**	**23**	**24**	**25**	**26**	**27**	**28**	**29**	**30**	**31**
**1**																															
**2**	0.000																														
**3**	0.721	0.721																													
**4**	0.000	0.000	0.721																												
**5**	0.687	0.687	0.582	0.687																											
**6**	0.705	0.705	0.705	0.705	0.842																										
**7**	0.674	0.674	0.984	0.674	0.942	0.00																									

**8**	0.663	0.663	0.086	0.663	0.533	0.806	0.973																								
**9**	0.613	0.613	0.034	0.613	0.438	0.770	0.993	0.006																							
**10**	0.585	0.585	0.585	0.585	0.263	0.596	0.518	0.138	0.280																						
**11**	0.905	0.905	0.00	0.905	0.611	0.608	0.878	0.035	0.034	0.455																					
**12**	0.459	0.459	0.943	0.459	0.666	0.006	0.005	0.819	0.726	0.241	0.820																				
**13**	0.379	0.379	0.529	0.379	0.563	0.994	0.929	0.334	0.630	0.094	0.759	0.627																			
**14**	0.311	0.311	0.707	0.311	0.301	0.811	0.775	0.657	0.399	0.912	0.644	0.810	0.347																		
**15**	0.179	0.179	0.425	0.179	0.869	0.429	0.524	0.032	0.196	0.255	0.210	0.667	0.139	0.834																	
**16**	0.631	0.631	0.035	0.631	0.751	0.853	0.634	0.199	0.304	0.646	0.022	0.928	0.561	0.235	0.308																
**17**	0.536	0.536	0.053	0.536	0.652	0.248	0.120	0.152	0.092	0.333	0.086	0.152	0.742	0.831	0.767	0.079															
**18**	0.500	0.500	0.068	0.500	0.321	0.665	0.879	0.017	0.000	0.408	0.075	0.824	0.635	0.233	0.256	0.485	0.165														
**19**	0.883	0.883	0.523	0.883	0.320	0.003	0.008	0.553	0.477	0.480	0.454	0.017	0.910	0.512	0.416	0.948	0.472	0.354													
**20**	0.768	0.768	0.713	0.768	0.409	0.666	0.598	0.352	0.465	0.028	0.636	0.389	0.341	0.891	0.809	0.908	0.489	0.535	0.635												
**21**	0.000	0.000	0.920	0.000	0.962	0.512	0.539	0.416	0.928	0.617	0.560	0.325	0.481	0.355	0.103	0.437	0.708	0.810	0.589	0.766											
**22**	0.901	0.901	0.350	0.901	0.143	0.343	0.262	0.415	0.485	0.028	0.343	0.158	0.664	0.322	0.646	0.252	0.133	0.724	0.192	0.181	0.958										
**23**	0.539	0.539	0.670	0.539	0.216	0.534	0.487	0.265	0.465	0.021	0.539	0.255	0.145	0.745	0.164	0.374	0.387	0.666	0.339	0.433	0.634	.024									
**24**	0.095	0.095	0.678	0.095	0.899	0.032	0.042	0.642	0.992	0.782	0.443	0.010	0.964	0.492	0.191	0.562	0.385	0.966	0.072	0.723	0.038	0.587	0.838								
**25**	0.164	0.164	0.922	0.164	0.781	0.163	0.144	0.548	0.916	0.355	0.725	0.336	0.636	0.268	0.430	0.178	0.365	0.682	0.158	0.782	0.227	0.243	0.158	0.965							

**26**	0.040	0.040	0.583	0.040	0.647	0.273	0.302	0.799	0.537	0.394	0.842	0.548	0.438	0.294	0.535	0.579	0.870	0.386	0.226	0.576	0.099	0.485	0.336	0.843	0.006						
**27**	0.577	0.577	0.577	0.577	0.324	0.554	0.510	0.896	0.711	0.762	0.557	0.736	0.570	0.037	0.815	0.086	0.499	0.466	0.306	0.667	0.659	0.149	0.233	0.841	0.131	0.384					
**28**	0.098	0.098	0.282	0.098	0.944	0.768	0.613	0.063	0.366	0.170	0.125	0.815	0.523	0.375	0.077	0.059	0.245	0.550	0.852	0.406	0.053	0.245	0.189	0.430	0.038	0.084	0.370				
**29**	0.948	0.948	0.664	0.948	0.527	0.685	0.608	0.363	0.374	0.053	0.641	0.362	0.408	0.895	0.942	0.801	0.448	0.407	0.711	0.000	0.980	0.244	0.562	0.581	0.984	0.828	0.495	0.580			
**30**	0.602	0.602	0.570	0.602	0.253	0.577	0.502	0.136	0.273	0.000	0.444	0.220	0.087	0.919	0.196	0.546	0.313	0.412	0.443	0.087	0.646	0.020	0.004	0.773	0.294	0.399	0.598	0.167	0.133		
**31**	0.654	0.654	0.003	0.654	0.457	0.782	0.946	0.013	0.001	0.370	0.004	0.816	0.986	0.753	0.243	0.110	0.043	0.004	0.513	0.573	0.998	0.385	0.497	0.882	0.947	0.577	0.937	0.275	0.497	0.352	

[a]: Data show the Pearson Correlation Coefficients between the parameters.

**. Correlation is significant at the 0.01 level (2-tailed).

*. Correlation is significant at the 0.05 level (2-tailed).

1: Methional, 2: 3-Methyl-4,5-dihydrothiophene, 3: 2-Methyl,4-propylthiazole, 4: p-(Methylthio) benzaldehyde, 5: 1-Octen-3-ol, 6: 3-Octanol, 7: Octanol, 8: Nonanol, 9: (E)-3-Decen-1-ol, 10: Ethyl phenylacetate, 11: Bornyl acetate, 12: Hexanal, 13: Heptanal, 14: 2-Methylene-hekzanal, 15: Octanal, 16: [E]-2-Octenal, 17: [E]-2-Nonenal, 18: 6-Methyl-5-heptene-2-one, 19: 3-Octanon, 20: Benzaldehyde, 21: Phenylacetaldehyde, 22: α-Pinene, 23: β-Pinene, 24: p-cymene, 25: Limonene, 26: Eucalyptol, 27: γ-Terpinene, 28: β-Linalool, 29: Thymol, 30: Carvacrol, 31: Geranylacetone.
